# The Choice of Screening Instrument Matters: The Case of Problematic Cannabis Use Screening in Spanish Population of Adolescents

**DOI:** 10.1155/2013/723131

**Published:** 2013-11-27

**Authors:** Danica Thanki, Antónia Domingo-Salvany, Gregorio Barrio Anta, Amparo Sánchez Mañez, Noelia Llorens Aleixandre, Josep Maria Suelves, Begoña Brime Beteta, Julián Vicente

**Affiliations:** ^1^Prevalence and Consequences and Data Management Unit, European Monitoring Centre for Drugs and Drug Addiction (EMCDDA), Cais do Sodré, 1249-289 Lisbon, Portugal; ^2^IMIM-Hospital del Mar, CIBERESP, Dr. Aiguader 88, 08003 Barcelona, Spain; ^3^Escuela Nacional de Sanidad, Instituto de Salud Carlos III, CIBERESP, Avenida Monforte de Lemos 5, 28029 Madrid, Spain; ^4^Unidad de Conductas Adictivas, Departamento de Salud Valencia, Hospital Arnau de Villanova, Plaza Clot de Joan s/n, Paterna, 46980 Valencia, Spain; ^5^Delegación del Gobierno para el Plan Nacional sobre Drogas (DGPNSD), Ministerio de Sanidad, Servicios Sociales e Igualdad, Plaza de España 17, 28071 Madrid, Spain; ^6^Agència de Salut Pública de Catalunya, Departament de Salut de la Generalitat de Catalunya, Roc Boronat 81-95, 08005 Barcelona, Spain

## Abstract

The aim of this study was to examine the feasibility of problem cannabis use screening instruments administration within wide school surveys, their psychometric properties, overlaps, and relationships with other variables. Students from 7 Spanish regions, aged 14–18, who attended secondary schools were sampled by two-stage cluster sampling (net sample 14,589). Standardized, anonymous questionnaire including DSM-IV cannabis abuse criteria, Cannabis Abuse Screening Test (CAST), and Severity of Dependence Scale (SDS) was self-completed with paper and pencil in the selected classrooms. Data was analysed using classical psychometric theory, bivariate tests, and multinomial logistic regression analysis. Not responding to instruments' items (10.5–12.3%) was associated with reporting less frequent cannabis use. The instruments overlapped partially, with 16.1% of positives being positive on all three. SDS was more likely to identify younger users with lower frequency of use who thought habitual cannabis use posed a considerable problem. CAST positivity was associated with frequent cannabis use and related problems. It is feasible to use short psychometric scales in wide school surveys, but one must carefully choose the screening instrument, as different instruments identify different groups of users. These may correspond to different types of problematic cannabis use; however, measurement bias seems to play a role too.

## 1. Introduction

Cannabis is the most widely used illicit drug worldwide [1]. Spain is currently the country with the highest last month prevalence of cannabis use among those aged 15–24 in Europe, with consistently high figures since 2005 (17.2–18.6%, [2]) and with the highest prevalence of regular (3–39 times within the past 12 months) and heavy (40 times or more within the past 12 months) use according to 2009/2010 HBSC (Health Behaviour in School-Aged Children, [3]) survey of 15-16-year olds [4].

A range of negative effects of regular cannabis use on adolescent health and psychosocial status have been identified, including adverse effects on psychosocial development and mental health (including developing psychotic symptoms), decrease in academic performance (which can lead to academic failure), and other negative outcomes later in life [5–9]. Since 2009, cannabis as a primary drug has overtaken heroin among those requesting drug treatment for the first time in their lives in the European Union. Its mention as a primary drug further increased in 2010 (accounting for more than 100 000 treatment demands). In this year, 76% of reported treatment entrants aged 15–19 years cited cannabis as their primary drug as did 86% of those younger than 15 years [10].

Chiefly from the perspective of planning public health interventions, like secondary/targeted drug prevention or drug treatment, there is considerable value in implementing screening instruments that are capable of detecting (probable) cannabis dependence or problematic use at the population level. Population or youth surveys in several European countries have recently started to incorporate screening instruments to measure cannabis dependence or problematic cannabis use [11–17]. In order to better understand these instruments, the European Monitoring Centre for Drugs and Drug Addiction (EMCDDA) has recommended methodological studies [18]. 

In the context of general population or school surveys, there is often shortage of space for inclusion of new items, as this may considerably increase the cost of the survey. Moreover, studies confirm that well-constructed short screening scales can be strong predictors of the same results found in more lengthy instruments or interviews [19, 20]. 

There are few epidemiological studies conducted in Europe to date applying psychometric scales to assess problematic forms of cannabis use in the general population of adolescents [13, 15–17]. Moreover, the relationship between different instruments has not been systematically studied.

The aims of the present study were (1) to explore the feasibility of administration of short cannabis instruments within a wide school survey of Spanish adolescents, (2) to obtain insight into the psychometric performance of CAST, SDS, and DSM-IV cannabis abuse criteria, (3) to find out whether the instruments identify the same groups of cannabis users by exploring their overlaps and associations of positivity on them with key variables.

## 2. Methods

Data collection was carried out on a sample of Spanish students within a biennial national school survey conducted by the Spanish National Programme on Drugs (DGPNSD) in 2006.

### 2.1. Reference Population and Sampling Frame

The reference population for this survey was students from 7 Spanish regions, selected by convenience, between the ages of 14 and 18 years who attended secondary schools. It was estimated that 82% of all Spanish youth of this age was schooled.

### 2.2. Sample

Two-stage cluster sampling was used, by randomly selecting 322 schools as first stage units and 644 classrooms as second stage units. In order to select the schools, the sampling frame was firstly stratified by autonomous region (seven strata) and school type (two strata, public and private schools). 9.6% of the registered students did not attend class on the date and time of the survey. 23.6% of schools and 3.5% of the classrooms were replaced because of refusal. 0.2% of students refused to participate in the study. The final sample consisted of 14,589 school attendees born between 1987 and 1992. Mean age was 15.6 years, and 46% were boys. The numbers of cannabis users in the studied sample were: lifetime use *n* = 5002, last 12 months use *n* = 4089, and last 30 days use *n* = 2735. 

### 2.3. Consent to Participate in the Study

The participation of students in the survey was based on a passive parental consent. Parents' associations of schools, school administrations, and regional educational authorities were informed about the nature, objectives, and characteristics of the study. All selected students were informed that participation in the survey was voluntary. To ensure confidentiality, questionnaires were anonymous.

### 2.4. Data Collection Tool

A standardized questionnaire was self-completed with paper and pencil by all of the students in the selected classrooms during a normal class (45–60 minutes), in the presence of the teacher who remained at the lectern throughout. The questionnaire included questions related to sociodemographic characteristics, drug use, perception of risk of different drug use behaviours, leisure time, level of perceived availability of different psychoactive drugs, social and health-related problems, sources of information on drugs, drug use by friends and classmates, and the attitudes of parents toward drug use. Frequency of cannabis use in the last 12 months and in the last 30 days was measured in categories of 0, 1, 2, 3, 4-5, 6–9, 10–19, 20–39, and 40 or more days (in the case of last 12 months use). Alcohol bingeing in the past 30 days was defined as drinking 5 or more glasses of alcohol in one single occasion.

Short cannabis disorders screening instruments were sought, which have been tested in similar settings [21]. Three, the shortest, were included in the questionnaire. All three were previously validated for self-administration.

1. DSM-IV cannabis abuse criteria: a 5-item instrument (corresponding to four abuse criteria), which has been incorporated in the US national survey on drugs since the year 2000 and administered in those who used the respective substance on 6 or more days in the past year [22]. By fulfilling at least one of the four abuse criteria, a person meets the criteria for abuse, which is diagnosed in the absence of dependence (DSM-IV, [23]). For the purpose of the present epidemiological survey, abuse criteria are used without excluding dependence cases.
2. The Severity of Dependence Scale (SDS): a 5-item scale that measures psychological components of dependence [24]. It has been successfully used to assess cannabis dependence in Germany, Australia, and Brazil [11, 25, 26], including adolescent populations of cannabis users, where a cut-off point of 4 for dependence was established [27].
3. The Cannabis Abuse Screening Test (CAST) is a 6-item scale screening for problematic forms of cannabis use, validated using DSM-IV cannabis dependence and cannabis use disorders criteria as a gold standard. It has been used successfully with teenagers, and a cutoff of 2 has recently been proposed to screen for either cannabis dependence or cannabis use disorders in this population [15]. Binary scoring procedure was used, as the resulting total score has a more intuitive interpretation, while the full scoring offers only modest measurement advantages [15].

See Table 1 for all instruments' items, answer options, and the respective scoring.

The instruments were adapted into Spanish and Catalan in a process including translation, back-translation, cognitive debriefing with young cannabis users, and discussion at an expert meeting. They were placed at the end of the questionnaire and addressed only to those adolescents who had indicated that they had used cannabis in the past 12 months. The assessment frame of all three instruments was “last 12 months.”

In the case of one missing item on a particular scale, in all analyses that included positivity on the instrument, its value was replaced by mean score of the rest of the scale items. This strategy was not applied to DSM-IV abuse criteria.

### 2.5. Data Analysis

All analyses were carried out using SPSS for Windows 16.0.1. 

Analyses of missing items and their relationship with gender, age, and frequency of use in the past year and past month were performed using the Chi-Square test.

An analysis of psychometric properties of the three cannabis screening instruments was carried out including item analysis. Unidimensionality was explored by principal components analysis and internal consistency measured by Cronbach alpha. Internal consistency was assessed by age, to examine possible age-related lack in response consistency. 

Overlap between the different instruments was examined. Instruments' total scores were tested for their relationship with frequency of cannabis use in the past 12 months. In this correlation analysis, averages of the above-mentioned categories were used to compute the Pearson coefficient and order of these categories to compute Spearman's rho.

A series of bivariate analyses (Chi-Square Test and One-Way ANOVA) and a multinomial logistic regression analysis was run to explore the associations of being positive/negative on the different combinations of instruments (in mutually exclusive categories) with key demographic, substance use, and drug-related problems variables. Students who were negative on all instruments were referent category. 

## 3. Results

### 3.1. Missing Items Analysis

Of the 4089 cannabis users in the past 12 months, 3569 fully completed the CAST scale, 3546 the SDS scale, and 3573 the DSM-IV abuse criteria. Between 10.5% and 12.3% of answers per item were missing (in most of these cases the entire scale was left blank). Leaving blank a substantial number of items was associated with reporting lower frequency of cannabis use in the past 12 months and in the past 30 days (Chi-Square Test, *P* < 0.001 in both cases). There were, however, 136 students who reported that they had used cannabis on at least 20 days during the past year, and their total scores of at least one scale could not be computed due to missing items. They represented 3% of all past year users. No association between the number of missing items and gender or age was found.

### 3.2. Psychometric Properties of the Instruments

Table 1 shows results of item analysis as well as some results for the composite scores of the instruments. Some items had a higher share in the total score than others: the first two items in the case of DSM-IV abuse, the third item of CAST, and item 4 of SDS. The importance of these items was generally confirmed by analysis of proportion positive if the item was deleted.

Principal components analysis revealed unidimensionality in each of the instruments with relatively high factor loadings. Less than 50% of variance was explained by the first component in all cases (46-47%). Cronbach alphas of the three instruments were moderate to satisfactory (0.613–0.762). The only item which would provide (small) improvement of alpha when deleted was item 4 of the SDS scale. 

Analysis of internal consistency by age group did not reveal a clear age-related pattern with the exception of CAST, where those aged 14 showed a somewhat lower internal consistency (results not shown). 

### 3.3. Positivity on the Instruments according to Published Cut-Off Points

At least one DSM-IV criterion for abuse was fulfilled by 28.6% (1023) of the instrument completers. 34.3% (1273) of students who completed at least five items of CAST had 2 or more points and would thus be assigned possible cannabis dependence/cannabis use disorders. SDS positive cases (possible dependence on cannabis) at a cutoff of 4 points or more were 16.3% (592). If only those, who used cannabis on six or more days in the past twelve months were considered for positivity, the figures would be 21.6%, 28.7%, and 11.7%, respectively. Students positive on the respective instruments constituted 7.0%, 8.7%, and 4.1% of the entire sample, or 5.3, 7.2, and 2.9%, if six or more days users only were considered.

Sensitivity analysis was performed taking into account the 136 daily or near-daily users whose total score could not be computed due to missing items. Assuming that all of these frequent users would cross the cut-off point on the respective instruments, the original range of prevalence estimates based on crossing the thresholds for positivity of the different instruments (see above) would change slightly to 2.9–7.9%. 

### 3.4. Relationships between Instruments

**Figure 1:**
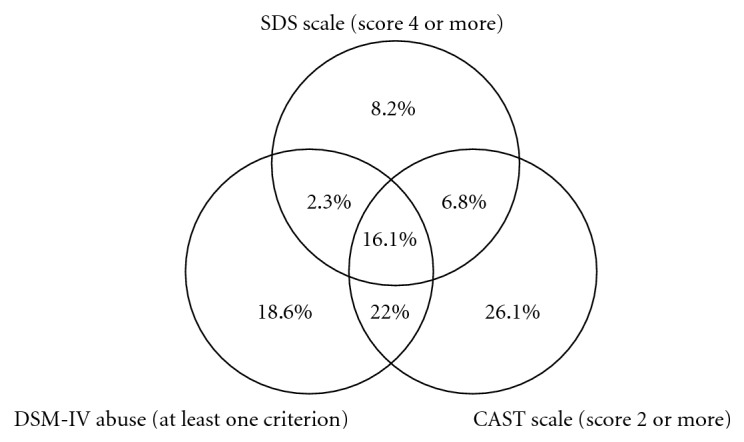
Overlap between the three studied instruments. Total percentages out of those positive on at least one scale (*n* = 1708).

Only 16.1% of all positives were positive on all three instruments. Out of the 1708 positive on at least one instrument, most cases, 70.9%, were positive on CAST criteria with 26.1% only on CAST; the second largest group, 59%, was positive on the DSM-IV abuse with 18.6% only on DSM-IV abuse. A smaller proportion, 33.4%, was positive on the SDS scale with 8.2% only on SDS (see Figure 1). 

Pearson correlations between the instruments' total scores were moderate: 0.363 between DSM-IV abuse and SDS, 0.456 between CAST and SDS, and 0.500 between CAST and DSM-IV abuse (*R* ^2^  between 0.13 and 0.25). 

### 3.5. Relationships of Scores and Positivity on Instruments with Frequency of Cannabis Use in the Past Twelve Months and Other Variables

CAST total score had the highest correlation of all the instruments with frequency of cannabis use in the past twelve months (0.567 and 0.588, Spearman and Pearson correlation coefficient, resp.). Number of fulfilled DSM-IV abuse criteria correlated with frequency of use in the past year only moderately (0.326 and 0.339, correlations in the same order), and SDS's total score correlations were the lowest (0.283 and 0.251). 

In bivariate analyses, there were significant associations between the mutually exclusive categories of all combinations of positivity/lack of positivity on the tested instruments and all of the following variables: years of cannabis use, gender, frequency of cannabis use in the past 30 days and in the past year, friends' consumption of cannabis, having an accident requiring medical help in the past 12 months within 6 hours after cannabis use, being under police arrest in the past 12 months within 6 hours after cannabis use, thinking that habitual cannabis smoking causes “quite some problems” or “a lot of problems,” smoking tobacco on some days a week or more during the past month, age at survey, not going to school for two or more days in the past month, because of not feeling like to, and two or more days of alcohol bingeing in the past month and going out one or more nights per week (see Table 2). 

In a multinomial logistic regression analysis (see Table 3), those being positive only on SDS were not significantly different from the referent category (negative on all instruments), with the exception of a higher proportion of those thinking that habitual cannabis use poses “quite some problems” or “a lot of problems” and a significantly higher proportion of those belonging to the youngest age group (14-15 years old). 

Out of the remaining categories of instrument positivity, those categories, which contained positivity on CAST (positive only on CAST or in combination with SDS, DSM-IV abuse or both) had the highest average number of years of cannabis use. They also had a higher prevalence of truancy, a higher prevalence of frequent cannabis use in the past month (10 or more days of use), higher, but mostly not statistically significant, prevalence of alcohol bingeing in the past month, a higher proportion of the majority (or all) friends consuming cannabis, and the highest prevalence of regular tobacco smoking in the past month (at least some days a week). Students who were positive on CAST also had a higher prevalence of suffering an accident requiring medical help and/or detention by police within six hours after cannabis use in the past year, but this was further strengthened if positivity on CAST was combined with positivity on DSM-IV abuse.

## 4. Discussion

The present study confirmed that it was feasible to use short psychometric instruments for problematic forms of cannabis use in a large national probabilistic survey on substance use in Spanish adolescents. Even though the instruments were placed at the end of the questionnaire, 88–90% of items were filled in. Missing answers occurred more often in individuals who reported low frequency of cannabis use. Sensitivity analysis was performed which did not find a significant influence of missing scale results in those reporting frequent cannabis use on the overall prevalence estimate. 

We have studied overlap between the three instruments and found that it was relatively small (16.1%) with more than half of the respondents positive on only one instrument. The instruments' total scores did not correlate highly either. This is partially due to the different concepts of problem cannabis use, which are behind the three instruments. The expected relationships in this respect would be a high overlap between DSM-IV abuse and CAST (the latter designed to screen for cannabis use disorders, that is, cannabis abuse and/or cannabis dependence) and a subgroup of abuse and/or CAST positive persons being identified as cannabis dependent by SDS. However, this was clearly not the case.

Moreover, focusing only on cannabis dependence gives a result which is similar to comparing all three instruments. If we were to explore the overlap between CAST and SDS, both validated against gold standard representing cannabis dependence, in the present study, we would still find only 28.1% of cases identified by both instruments, while almost 60% would be positive only according to CAST and 12.9% only according to SDS. Swift et al. [28] studied three measures of cannabis dependence, among them the SDS and found 56% of cases identified by all three measures.

One possible explanation of low overlap between screening instruments in the present study might be the existence of different types of problematic cannabis users, with different characteristics and different kinds of problems experienced by them [29]. Another possibility is the presence of measurement bias, with varying extent in the three studied instruments. The multinomial logistic regression analysis discussed sheds some light on this possibility.

According to the results of this analysis, students positive only on SDS scale were similar to those who were negative on all instruments with the exception of being a younger age and more likely to think that habitual cannabis use causes “quite some problems” or “a lot of problems”. On the other hand, those groups which included positivity on CAST had the longest history of cannabis use, higher prevalence of different problems, some of which were directly related to cannabis use. In this group, there was a lower proportion of girls, higher tobacco smoking, markedly higher prevalence of high frequency cannabis use in the past month, more of their friends smoked cannabis, while they thought less that habitual cannabis use caused substantial problems. DSM-IV abuse positivity has further strengthened CAST's association with such problems as being detained by the police or having an accident shortly after cannabis use. This is consistent with two abuse criteria—legal problems and using the substance in physically hazardous situations—and confirms concurrent validity of the instrument. Fulfilling DSM-IV abuse criteria was associated less strongly than CAST with history and frequency of cannabis use, and other problems potentially related to cannabis use, but more strongly than SDS positivity.

This suggests that the usefulness of SDS in screening for problematic cannabis use in the general population of adolescents is limited, as it may identify a considerable proportion of “wrong people.” This finding from the multinomial logistic regression analysis is further corroborated by the weak association of the scale with frequency of use in the past year—an important indicator within the diagnostic approach [30, 31] and by the fact that item 4 of the scale (wish to stop) contributed to the total score considerably while its removal would improve the internal consistency of the instrument. This weaker performance might be associated with the concept behind the scale, namely psychological components of dependence. These might be difficult to assess or confusing for adolescents. For example, other authors have found a response bias in adolescents in the direction of overreporting dependence, mainly its symptoms “being unable to cut down” and “tolerance” [32]. Another study suggested that substance use disorders diagnostic criteria might have different meaning for adolescents, who may experience rapid development of tolerance after initiation of use and may be confused about a question on “using more or longer than intended” as they typically do not foresee a limit of consumption but intend to become intoxicated [33].

Although conducted on a large probabilistic sample, the present study has several limitations. The scales administration was based on self-assessment, moreover of relatively complex concepts. The sampling frame of the present study was adolescents aged 14–18 who were schooled; 18% of this age group were estimated to be unschooled and thus outside of the sampling frame. Moreover, almost 10% of the students of the selected classrooms were not attending school on the day of survey. There are associations of dropping out of school and truancy with substance use and related problems shown by numerous studies (e.g., [34] and [16, page 172]). However, the analysis of psychometric properties of the explored instruments may be less biased by these factors than, for example, prevalence estimation.

The placing of the screening instruments (almost at the end of the questionnaire), and, possibly, the order of their presentation may have affected the number of missing responses and/or the reliability of responses due to such factors as running out of time, or tiredness of the respondent and possibly the measures of psychometric properties of the instruments. A study comparing the effects of different orders of presentation would be necessary for clarification of this question.

Furthermore, as establishing clinical diagnoses was not part of this study, its findings have to be interpreted cautiously in relation to these concepts. The reported psychometric properties of the applied instruments might not be transferable to clinical or targeted samples of heavy cannabis users, owing to “spectrum bias” [35] in those samples. Nevertheless, it is important to know how instruments to detect problematic cannabis use perform in the general adolescent population.

In conclusion, SDS, CAST, and DSM-IV abuse criteria have performed moderately well in the present sample, but identified largely different groups of users with only modest overlaps, even between scales validated against the same gold standard. Given the fact that a researcher planning an epidemiological survey may decide to choose any of these instruments to represent a measure of “problematic cannabis use,” this finding may be highly relevant. While one possible explanation for these differences might be the existence of different subtypes of problematic cannabis use, measurement bias of varying extent may also play a role.

The present analyses put in question the validity of the SDS scale in the general adolescent population. The concept of psychological components of dependence represented by the scale may be difficult to understand for adolescent cannabis users. This may be the reason for high scoring on SDS items operationalizing this concept in the youngest lower frequency users who have few cannabis-related problems and who think that habitual cannabis use causes “quite some problems” or “a lot of problems”.

On the other hand, CAST scale had generally strong relationship with frequency of cannabis use in the last year and other variables suggesting its concurrent validity. DSM-IV abuse was less strongly associated with intensive and long-term use, but its association with legal problems and an item indicating possible use in physically hazardous situations corroborated the validity of the instrument in relation to the concept behind it.

The findings of the present study are relevant also from the broader perspective of using short, self-report-based screeners of complex, more abstract concepts in the population of adolescents in psychiatry, general practice, and clinical psychology.

Further research, especially qualitative, is needed to shed light on adolescents' understanding of psychometric instruments' items related to cannabis dependence symptoms in order to improve the ascertainment of problematic cannabis use in this population.

## Figures and Tables

**Table:** (a) DSM-IV abuse criteria

Item	Item basic statistics		Importance of the single item (difficulty)		Internal consistency and discriminative power			
	Mean score (points)	SD (points)	Mean score as percent of mean total score	Frequency of high scoring (%)	Positive on scale if item deleted (%)	Factor loading (CFA)	Cronbach alpha if deleted	Item correlation /discrimination^a^
DSM-IV criteria for abuse: mean total score: 0.46, SD 0.87; Cronbach alpha of scale: 0.613. In principal components analysis, 46.9% variance explained by 1st factor with eigenvalue 1.875.								

DSM-1	Sometimes people who use cannabis have serious problems at home, work, or school—such as (i) neglecting their children, (ii) missing work, or school, (iii) doing a poor job at work or school, (iv) losing a job or dropping out of school During the past 12 months, did using cannabis cause you to have serious problems like this either at home, work, or school?							
	0.15	0.36	32.61	n.a.	21.8	0.685	0.545	0.540
DSM-2	During the past 12 months, did you regularly use cannabis and then do something where using cannabis might have put you in physical danger?							
	0.14	0.35	30.43	n.a.	22.7	0.653	0.563	0.483
DSM-3	During the past 12 months, did using cannabis cause you to do things that repeatedly got you in trouble with the law?							
	0.09	0.29	19.57	n.a.	26.5	0.705	0.526	0.322
DSM-4	During the past 12 months, did you have any problems with family or friends that were probably caused by your use of cannabis?							
	0.12	0.32	n.a.	n.a.	n.a.	n.a.	n.a.	n.a.
DSM-5	Did you continue to use cannabis even though you thought it caused problems with family or friends?							
	0.16	0.36	n.a.	n.a.	n.a.	n.a.	n.a.	n.a.
DSM-4+5	The last two items combined to obtain fourth DSM-IV criterion for abuse							
	0.08	0.27	17.39	n.a.	26.7	0.694	0.537	0.274

^a^For DSM-IV criteria: proportion of those positive for abuse (at least one criterion fulfilled) who answered the item positively; for CAST: proportion of those with total score 2+ (34.1% of the students fully filling the scale) who had a score of 1 on the item; SDS: item-total correlation.

**Table:** (b) CAST

Item	Item basic statistics		Importance of the single item (difficulty)		Internal consistency and discriminative power			
	Mean score (points)	SD (points)	Mean score as percent of mean total score	Frequency of high scoring (%)	Positive on scale if item deleted (%)	Factor loading (PCA)	Cronbach alpha if deleted	Item correlation/ discrimination
Cannabis abuse screening test (CAST) scale: mean total score: 1.33 (1.35), SD 1.68 (1.69); Cronbach alpha of scale: 0.762. In principal components analysis, 46.1% variance explained by 1st factor with eigenvalue 2.76.								

	Has the following happened to you during the last 12 months?							
CAST-1	(a) Have you ever smoked cannabis before midday? 0 = never; 0 = rarely; 1 = from time to time; 1 = fairly often; 1 = very often							
	0.24	0.43	18.1	n.a.	29.4	0.722	0.717	0.624
CAST-2	(b) Have you ever smoked cannabis when you were alone? 0 = never; 0 = rarely; 1 = from time to time; 1 = fairly often; 1 = very often							
	0.21	0.41	15.8	n.a.	30.7	0.717	0.718	0.549
CAST-3	(c) Have you ever had memory problems when you smoked cannabis? 0 = never; 1 = rarely; 1 = from time to time; 1 = fairly often; 1 = very often							
	0.32	0.47	24.1	n.a.	28.1	0.701	0.722	0.732
CAST-4	(d) Have friends or members of your family ever told you that you ought to reduce or stop your cannabis use? 0 = never; 1 = rarely; 1 = from time to time; 1 = fairly often; 1 = very often							
	0.20	0.40	15.0	n.a.	31.4	0.726	0.714	0.537
CAST-5	(e) Have you ever tried to reduce or stop your cannabis use without succeeding? 0 = never; 1 = rarely; 1 = from time to time; 1 = fairly often; 1 = very often							
	0.21	0.40	15.8	n.a.	30.4	0.542	0.759	0.472
CAST-6	(f) Have you ever had problems because of your use of cannabis (argument, fight, accident, bad result at school, etc.)? 0 = never; 1 = rarely; 1 = from time to time; 1 = fairly often; 1 = very often							
	0.17	0.37	12.8	n.a.	31.6	0.645	0.735	0.434

Note: Figures in parentheses relate to calculations after replacing missing values. Displayed only if different from those obtained without applying this strategy.

**Table:** (c) SDS

Item	Item basic statistics		Importance of the single item (difficulty)		Internal consistency and discriminative power			
	Mean score (points)	SD (points)	Mean score as percent of mean total score	Frequency of high scoring (%)	Positive on scale if item deleted (%)	Factor loading (CFA)	Cronbach alpha if deleted	Item correlation/ discrimination
Severity of dependence scale (SDS): mean total score: 1.57 (1.56), SD 2.42 (2.42); Cronbach alpha of scale: 0.672. In principal components analysis, 45.6% variance explained by 1st factor with eigenvalue 2.28.								

SDS-1	In the last 12 months, (1) did you ever think your use of cannabis was out of control? 0 = never or almost never; 1 = sometimes; 2 = often; 3 = always or nearly always							
	0.28	0.73	17.83	4.7	12.6	0.67	0.61	0.67
SDS-2	(2) Did the prospect of missing a smoke make you very anxious or worried? 0 = never or almost never; 1 = sometimes; 2 = often; 3 = always or nearly always							
	0.23	0.58	14.65	1.9	13.6	0.757	0.596	0.678
SDS-3	(3) Did you worry about your use of cannabis? 0 = not at all; 1 = a little; 2 = quite a lot; 3 = a great deal							
	0.31	0.68	19.75	2.9	11.4	0.761	0.562	0.741
SDS-4	(4) Did you wish you could stop? 0 = never or almost never; 1 = sometimes; 2 = often; 3 = always or nearly always							
	0.51	0.98	32.48	10.3	9.7	0.563	0.680	0.683
SDS-5	(5) How difficult would you find it to stop or go without cannabis? 0 = not difficult; 1 = quite difficult; 2 = very difficult; 3 = impossible							
	0.24	0.65 (0.64)	15.29	2.8	13.3	0.602	0.655	0.566

Note: figures in parentheses relate to calculations after replacing missing values.

**Table 2:** Associations of different combinations of being positive on the studied instruments with key demographic, substance use, and drug-related problems variables. Distributions and means.

	All last year cannabis users with computable total scores	Negative on all three instruments	Positive only on CAST	Positive only on SDS	Positive on CAST and SDS	Positive only on DSM-IV abuse	Positive on DSM-IV abuse and CAST	Positive on DSM-IV abuse and SDS	Positive on all three instruments
	(*n* = 3516)	(*n* = 1808)	(*n* = 445)	(*n* = 140)	(*n* = 116)	(*n* = 318)	(*n* = 375)	(*n* = 39)	(*n* = 275)
Years since first cannabis use (mean (SD))	1.65 (1.43)	1.30 (1.25)	2.07 (1.40)	1.27 (1.10)	1.93 (1.50)	1.57 (1.39)	2.31 (1.55)	1.91 (1.44)	2.51 (1.71)
Percent of girls	51.4%	57.60%	46.70%	57.10%	57.80%	45.00%	39.50%	35.90%	38.50%
Two or more days of truancy in the past month (total *n*)^o^	11.7% (3090)	7.30% (1615)	15.00% (381)	5.60% (126)	8.40% (95)	14.80% (277)	20.10% (329)	14.30% (28)	25.10% (239)
Use of cannabis 10 days or more in the past month	18.6%	27.80%	38.00%	2.90%	38.80%	9.40%	41.90%	23.10%	55.60%
Last year cannabis use 1 to 3 days	34.60%	49.10%	12.60%	55.7%	13.80%	36.50%	8.30%	15.40%	10.20%
Last year cannabis use 40 days or more	22.70%	6.90%	41.30%	6.4%	44.00%	17.90%	51.70%	23.10%	61.80%
Two or more days of alcohol bingeing in the past month	48.4%	40.20%	55.70%	40.70%	51.70%	52.20%	63.50%	53.80%	67.30%
Majority or all friends consume cannabis (total *n*)^a^	40.9% (3491)	27.30% (1799)	56.70% (443)	28.10% (139)	56.1% (114)	38.50% (314)	64.20% (371)	59.00% (39)	73.50% (272)
Suffered an accident requiring medical help within six hours of cannabis use in the last twelve months	5.6%	1.20%	7.60%	1.40%	7.80%	4.10%	16.0%	10.30%	19.30%
Detained by police within six hours of cannabis use in the last twelve months	5.8%	1.00%	6.10%	2.10%	6.00%	5.00%	15.20%	7.70%	26.90%
Thinking that habitual cannabis use causes “quite some problems” or “a lot of problems” (total *n*)^a^	75.6% (3293)	83.20% (1700)	62.10% (412)	89.70% (126)	67.30% (107)	79.40% (296)	60.00% (360)	73.00% (37)	61.60% (255)
Smoked tobacco some days per week or more often in the past 30 days	61.10%	51.00%	73.50%	55.00%	75.90%	58.50%	80.30%	56.40%	81.10%
Going out one or more nights per week	68.5%	64.20%	76.40%	60.00%	71.60%	65.40%	76.50%	56.40%	81.50%
Aged 14-15	33.6%	34.20%	24.50%	47.90%	31.9%	38.70%	32.8%	33.30%	33.5%
Aged 16	28.1%	29.40%	27.90%	25.70%	25.00%	24.8%	26.4%	38.50%	26.5%
Aged 17-18	38.3%	36.30%	47.60%	26.40%	43.10%	36.5%	40.8%	28.20%	40.00%

Notes: alcohol bingeing was defined as drinking five or more glasses of alcohol at one single occasion. ^a^The total *n* in case of these variables is different, because answer category “I don't know” was treated in the analysis as a missing value (only valid percentages are reported). ^o^The total *n* in case of this variable is different, because other reasons of not attending class were excluded from the analysis and only valid percentage is reported.

**Table 3:** Results of a multinomial regression analysis comparing different combinations of being positive on the studied instruments with key demographic, substance use, and drug-related problems variables.

Variable instrument positivity	AOR^a^		95% CI
Two or more days of truancy in the past month (%)			
SDS only	0.80		0.34–1.89
CAST and SDS	0.82		0.36–1.87
CAST only	1.65∗		1.12–2.44
DSM-IV abuse only	2.05∗		1.35–3.12
DSM-IV abuse and CAST	2.08∗∗		1.40–3.09
DSM-IV abuse and SDS	2.19		0.71–6.81
DSM-IV abuse, CAST, and SDS	2.54∗∗		1.63–3.95
Use of cannabis 10 days or more in the past month (%)			
SDS only	0.58		0.17–1.91
CAST and SDS	6.21∗∗		3.54–10.90
CAST only	4.93∗∗		3.44–7.08
DSM-IV abuse only	1.18		0.68–2.04
DSM-IV abuse and CAST	5.15∗∗		3.53–7.50
DSM-IV abuse and SDS	3.58∗		1.23–10.41
DSM-IV abuse, CAST, and SDS	8.10∗∗		5.32–12.33
Two or more days of alcohol bingeing in the past month (%)			
SDS only	0.99		0.65–1.50
CAST and SDS	1.23		0.77–1.95
CAST only	1.11		0.86–1.45
DSM-IV abuse only	1.34∗		1.00–1.78
DSM-IV abuse and CAST	1.52∗∗		1.14–2.03
DSM-IV abuse and SDS	1.68		0.70–4.02
DSM-IV abuse, CAST, and SDS	1.62∗∗		1.14–2.32
Majority or all friends consume cannabis (%)			
SDS only	1.35		0.87–2.10
CAST and SDS	1.71∗		1.05–2.78
CAST only	2.02∗∗		1.54–2.66
DSM-IV abuse only	1.67∗∗		1.24–2.25
DSM-IV abuse and CAST	2.33∗∗		1.72–3.14
DSM-IV abuse and SDS	3.75∗∗		1.50–9.39
DSM-IV abuse, CAST, and SDS	3.07∗∗		2.11–4.49
Suffered an accident requiring medical help within six hours of cannabis use in the last twelve months (%)			
SDS only	1.63		0.37–7.18
CAST and SDS	2.42		0.84–7.00
CAST only	2.59∗∗		1.32–5.10
DSM-IV abuse only	2.27		0.99–5.22
DSM-IV abuse and CAST	5.62∗∗		2.99–10.54
DSM-IV abuse and SDS	3.81		0.78–18.54
DSM-IV abuse, CAST, and SDS	5.87∗∗		3.01–11.46
Detained by police within six hours of cannabis use in the last twelve months (%)			
SDS only	2.66		0.74–9.54
CAST and SDS	2.71		0.92–7.95
CAST only	3.12∗∗		1.55–6.28
DSM-IV abuse only	4.03∗∗		1.88–8.64
DSM-IV abuse and CAST	5.94∗∗		3.10–11.38
DSM-IV abuse and SDS	4.13		0.84–20.29
DSM-IV abuse, CAST, and SDS	9.48∗∗		4.87–18.46
Thinking that habitual cannabis use causes quite some problems or a lot of problems (%)			
SDS only	2.09∗		1.06–4.09
CAST and SDS	0.72		0.43–1.20
CAST only	0.60∗∗		0.45–0.81
DSM-IV abuse only	1.01		0.70–1.45
DSM-IV abuse and CAST	0.70∗		0.51–0.96
DSM-IV abuse and SDS	1.88		0.60–5.86
DSM-IV abuse, CAST, and SDS	0.82		0.56–1.12
Smoked tobacco some days per week or more often in the past month (%)			
SDS only	1.12		0.75–1.68
CAST and SDS	1.97∗		1.16–3.32
CAST only	1.72∗∗		1.30–2.27
DSM-IV abuse only	1.78		0.88–1.57
DSM-IV abuse and CAST	2.31∗∗		1.66–3.21
DSM-IV abuse and SDS	0.81		0.34–1.93
DSM-IV abuse, CAST, and SDS	1.78∗∗		1.20–2.64
Aged 14-15∗ (%)			
SDS only	1.95∗		1.14–3.33
CAST and SDS	1.06		0.58–1.93
CAST only	0.94		0.67–1.32
DSM-IV abuse only	1.52∗		1.06–2.19
DSM-IV abuse and CAST	1.95∗∗		1.35–2.81
DSM-IV abuse and SDS	3.64∗		1.20–10.97
DSM-IV abuse, CAST, and SDS	2.25∗∗		1.45–3.46
Aged 16∗ (%)			
SDS only	1.41		0.82–2.43
CAST and SDS	0.95		0.53–1.68
CAST only	0.95		0.69–1.31
DSM-IV abuse only	1.00		0.69–1.45
DSM-IV abuse and CAST	1.21		0.84–1.73
DSM-IV abuse and SDS	2.15		0.70–6.66
DSM-IV abuse, CAST, and SDS	1.39		0.90–2.13

Years since first cannabis use	Estimate (B)	SE	Wald

SDS only	0.05	0.10	0.28
CAST and SDS	0.23	0.10	5.41∗
CAST only	0.27	0.06	24.72∗∗
DSM-IV abuse only	0.16	0.07	5.82∗
DSM-IV abuse and CAST	0.40	0.06	48.63∗∗
DSM-IV abuse and SDS	0.36	0.14	6.49∗
DSM-IV abuse, CAST, and SDS	0.39	0.07	34.59∗∗

All of the displayed variables were entered in the multinomial logistic regression model. All of them were significant in the overall model.

Stars signify statistical significance in the multinomial logistic regression model (∗*P* < 0.05, ∗∗*P* < 0.01).

The reference category is “negative on all instruments.”

∗Compared to those aged 17-18.

## Abbreviations

EMCDDA: European Monitoring Centre for Drugs and Drug Addiction
UNODC: United Nations Office for Drugs and Crime
HBSC: Health Behaviour in School-Aged Children, World Health Organization's survey
WHO: World Health Organization
SDS: Severity of Dependence Scale
CAST: Cannabis Abuse Screening Test
DSM-IV: Diagnostic and Statistical Manual of Mental Disorders, 4th revision.
